# Germline Genetic Variants of the Renin-Angiotensin System, Hypoxia and Angiogenesis in Non-Small Cell Lung Cancer Progression: Discovery and Validation Studies

**DOI:** 10.3390/cancers12123834

**Published:** 2020-12-18

**Authors:** Maria Joana Catarata, Rui Medeiros, Maria José Oliveira, Alice Pêgo, João Gonçalo Frade, Maria Fátima Martins, Carlos Robalo Cordeiro, Felix J. F. Herth, Michael Thomas, Mark Kriegsmann, Michael Meister, Marc A. Schneider, Thomas Muley, Ricardo Ribeiro

**Affiliations:** 1 i3S-Institute for Research & Innovation in Health, University of Porto, 4200-135 Porto, Portugal; mariajo@ineb.up.pt (M.J.O.); ricardo.ribeiro@i3s.up.pt (R.R.); 2 INEB-Institute of Biomedical Engineering, University of Porto, 4200-135 Porto, Portugal; 3 Department of Pulmonology, Coimbra Hospital and University Center, 3000-075 Coimbra, Portugal; 6165@chuc.min-saude.pt (A.P.); crcordeiro@fmuc.uc.pt (C.R.C.); 4 Faculty of Medicine, University of Porto, 4200-319 Porto, Portugal; ruimedei@ipoporto.min-saude.pt; 5 Molecular Oncology and Viral Pathology Group-Research Centre, Portuguese Institute of Oncology, 4200-072 Porto, Portugal; 6 Department of Clinical Pathology, Coimbra Hospital and University Center, 3000-075 Coimbra, Portugal; joao.goncalo.frade@chuc.min-saude.pt (J.G.F.); mfmartins@chuc.min-saude.pt (M.F.M.); 7 Faculty of Medicine, University of Coimbra, 3004-504 Coimbra, Portugal; 8 Department of Pulmonology and Critical Care Medicine, Thoraxklinik, University of Heidelberg, 69126 Heidelberg, Germany; felix.herth@med.uni-heidelberg.de; 9 Translational Lung Research Centre (TLRC), member of the German Centre for Lung Research (DZL), 69120 Heidelberg, Germany; michael.thomas@med.uni-heidelberg.de (M.T.); mark.kriegsmann@med.uni-heidelberg.de (M.K.); michael.meister@med.uni-heidelberg.de (M.M.); Marc.Schneider@med.uni-heidelberg.de (M.A.S.); thomas.muley@med.uni-heidelberg.de (T.M.); 10 Department of Thoracic Oncology, Thoraxklinik, University of Heidelberg, 69126 Heidelberg, Germany; 11 Institute of Pathology, University Hospital Heidelberg, 69120 Heidelberg, Germany; 12 Translational Research Unit, Thoraxklinik, University of Heidelberg, 69126 Heidelberg, Germany; 13 Laboratory of Genetics and Institute of Environmental Health, Faculty of Medicine, University of Lisbon, 1649-026 Lisboa, Portugal

**Keywords:** genetic polymorphisms, lung cancer, renin–angiotensin, hypoxia, angiogenesis

## Abstract

**Simple Summary:**

The presence of polymorphic gene variants in the human genome provides extensive genetic (and eventually phenotypic) variation affecting both normal physiological mechanisms and cancer pathogenesis. Functional genetic polymorphisms might have predictive and/or prognostic value in lung cancer, opening novel opportunities to improve prediction and guide clinical reasoning and therapeutics in lung cancer patients. Recent knowledge pinpoints a pleiotropic role for renin-angiotensin system, particularly in the lung and mainly through locally regulated alternative molecules and secondary pathways. Dysregulation of this system play a role in cell proliferation, hypoxia and angiogenesis, which processes are involved in lung cancer progression. Here we suggest that polymorphic variants in genes coding for renin-angiotensin system might play a role in Non-Small Cell Lung Cancer progression.

**Abstract:**

Introduction: The renin–angiotensin system (RAS) is involved in cell proliferation, immunoinflammatory response, hypoxia and angiogenesis, which are critical biological processes in lung cancer. Our aim was to study the association of putatively functional genetic polymorphisms in genes coding for proteins involved in RAS, hypoxia and angiogenesis with non-small cell lung cancer (NSCLC) prognosis. Methods: Genotyping of 52 germline variants from genes of the RAS and hypoxic/angiogenic factors/receptors was performed using MassARRAY iPLEX Gold in a retrospective cohort (*n* = 167) of advanced NSCLC patients. Validation of the resulting genetic markers was conducted in an independent group (*n* = 190), matched by clinicopathological characteristics. Results: Multivariate analysis on the discovery set revealed that *MME* rs701109 C carriers were protected from disease progression in comparison with homozygous T (hazard ratio (HR) = 0.5, 95% confidence interval (CI) = 0.2–0.8, *p* = 0.010). Homozygous A and T genotypes for *KDR* rs1870377 were at increased risk for disease progression and death compared to heterozygous (HR = 1.7, 95% CI = 1.2–2.5, *p* = 0.005 and HR = 2.1, 95% CI = 1.2–3.4, *p* = 0.006, respectively). Carriers of homozygous genotypes for *ACE2* rs908004 presented increased risk for disease progression, only in the subgroup of patients without tumour actionable driver mutations (HR = 2.9, 95% CI = 1.3–6.3, *p* = 0.010). Importantly, the association of homozygous genotypes in *MME* rs701109 with risk for disease progression was confirmed after multivariate analysis in the validation set. Conclusion: This study provides evidence that *MME* polymorphism, which encodes neprilysin, may modulate progression-free survival in advanced NSCLC. Present genetic variation findings will foster basic, translational, and clinical research on their role in NSCLC.

## 1. Introduction

Lung cancer is one of the most common malignancies worldwide and the most common cause of cancer death, with over one million people diagnosed worldwide each year [1].

The renin–angiotensin system (RAS) is an established primary regulator of blood pressure, homeostasis, and natriuresis [2]. Recent evidences indicate that angiotensin peptides might have a role in tumour cell proliferation, inflammation, immune surveillance, hypoxia and angiogenesis, which have been recognized as hallmarks of cancer, and thereby actively participating in lung tumour microenvironment regulation [3,4]. The enzymatic cascade leading to Angiotensin II (Ang II) production includes renin, which cleaves angiotensinogen (AGT) to form angiotensin I (Ang I). Angiotensin-converting enzyme (ACE) cleaves Ang I to produce Ang II [2]. The chymase, which might catalyse the conversion from Ang I to Ang II, has been associated with activation of proliferation, adhesion, regulation of E-cadherin expression, and modulation of the immunosuppressive microenvironment in lung carcinoma [5,6]. Angiotensin-(1-7) (Ang-(1-7)) has opposite effects to Ang II through binding to Mas receptor (MasR), and is obtained by catalysis from the enzymes ACE type 2 (ACE2) or neprilysin (NEP) [7]. On its turn, NEP has been associated with some malignancies, such as prostate, renal and lung cancer [8].

In vitro and in vivo studies showed that activation of the ACE/Ang II/ angiotensin II type 1 receptor (ATR1) axis promotes cancer cell proliferation, invasion, angiogenesis and reduced immunosurveillance [9,10,11]. Conversely, anti-tumour effects were demonstrated for the ACE2/Ang-(1-7)/MasR axis [12,13,14]. Upregulated expression of *ATR1* transcripts in lung tumours suggests that Ang II, acting upon the ATR1, plays an important role in cell growth and invasion [15], whereas decreased expression of genes encoding for ACE2/Ang-(1–7)/MasR axis proteins in lung tumours halt MasR signalling, which is contrary to Ang II/ATR1 [16]. Notably, clinical studies revealed that RAS inhibitors combined with chemotherapy and Epidermal growth factor receptor (EGFR) tyrosine kinase inhibitors prolonged progression-free survival in non-small cell lung cancer (NSCLC) patients [17,18,19].

Activity and modulation of the RAS are dependable of germline genetic variations [20], somatic mutations [21], environmental stimulus and from hypoxic lung tumour microenvironment [22]. Additionally, the RAS is causally and consequently associated with hypoxia and angiogenesis, considered in continuum as branches of RAS [4]. Notwithstanding, the RAS remains largely unexplored at translational and clinical levels in cancer, particularly NSCLC. In this study, we sought to evaluate whether functional genetic variants coding for proteins of RAS and hypoxic/angiogenic factors/receptors have predictive and/or prognostic value in NSCLC using progression-free survival (PFS) and overall survival (OS) as clinical endpoints.

## 2. Material and Methods

### 2.1. Study Population: Discovery and Validation Sets

This study comprised a retrospective cohort of histologically confirmed NSCLC patients (*n* = 167) with clinical advanced stage at diagnosis (IIIA-IVB), between 2012 and 2018. Participants were consecutively recruited during routine follow-up visit, since August 2017 to October 2018 from Coimbra University Hospital (discovery set). The validation set comprised an independent cohort of NSCLC patients (*n* = 190) selected from the Lung Biobank Heidelberg, member of the Biomaterialbank Heidelberg (BMBH) and the biobank platform of the German Centre for Lung Research (DZL). All patients were recruited at Thoraxklinik (University Hospital of Heidelberg) between 2015–2018, using the same matched clinical and pathological criteria from the discovery set.

Histolopathogical diagnoses in both sets were made by experienced pathologists following the 2009 and 2015 WHO classification criteria for lung cancer, accordingly to the year of patient’s diagnosis. The existence of concomitant primary tumours in another organ was considered as exclusion criteria for both cohorts. Clinical data was retrieved from clinical charts on pathological background, previous medication, Eastern Cooperative Oncology Group performance status (ECOG PS) at diagnosis, TNM staging according to 8th classification, tumour mutational status, type of cancer treatment, disease progression/death by clinical authors. Informed consent was obtained from each participant and all procedures are in agreement with Helsinki Declaration.

In the discovery set, somatic mutations in *EGFR*, *ALK* and *ROS1* were determined using fluorescence in situ hybridization (FISH), in patients with adenocarcinoma stage IVA-IVB, whereas for the validation set, somatic mutations were determined through panel-based next generation sequencing in advanced NSCLC patients [23]. In both cohorts, targeted therapies were administered to carriers of actionable driver mutations, whereas checkpoint inhibitors were used as second-, or third-line therapy for patients with PD-L1 expression on tumour cells below 50%.

The primary endpoint was PFS and the time-to-disease progression was calculated in months, from the date of first line chemotherapy/target therapy, until the date of progression according to RECIST criteria. OS was included as secondary endpoint, and the time-to-death was computed in months from the date of first line chemotherapy/target therapy until the date of death/date of last visit.

### 2.2. Selection of Genetic Polymorphisms

Fifty-two single nucleotide polymorphisms (SNP) included in the present study from the genes *ACE*, *ACE2*, *AGT*, *AGTR1*, *AGTR2*, *MME*, *CMA1*, *MAS1*, *HIF1A*, *VEGFA*, *KDR*, *PGF* and *FLT1* (Table S1) were selected from search in genomic public databases, in silico analysis and review of scientific literature, to identify putatively functional polymorphisms [24,25,26,27,28,29,30,31,32,33,34,35,36,37]. Polymorphisms with minor allele frequency below 1% were excluded [37].

### 2.3. Genotyping

#### 2.3.1. Discovery Set

Each patient donated a sample of blood (~4 mL) for research, collected to EDTA-Vacutainer tubes, at the same time of blood collection for routine analytic follow-up. The collected blood was centrifuged to obtain the buffy coat, which was used to isolate and purify DNA by the EZ1 DNA Blood kit in the EZ1 BioRobot (QIAgen, Hilden, Germany). The selected SNPs were genotyped using the Sequenom Mass ARRAY matrix-assisted laser desorption/ionization time-of-flight mass spectrometry platform (Sequenom, San Diego, CA, USA). Primers were designed using semi-automated Assay Design 3.1 Software (Sequenom). The global genotyping success rate was 99.85% with 100% replication agreement among one third of the samples.

#### 2.3.2. Validation Set

Buffy coat samples were provided by Lung Biobank Heidelberg from patients diagnosed with advanced NSCLC (clinical stage IIIA-IVB). DNA extraction and purification were conducted using the FlexiGene DNA Kit (QIAgen). The resulting genetic polymorphisms, associated with disease progression or overall survival from multivariate analysis in the discovery set, were genotyped in the validation set using Real-Time PCR. Allelic discrimination was obtained using TaqMan SNP genotyping assays: *ACE2* rs908004 (Assay ID: C___8816953_20), *MME* rs701109 (Assay ID: C____964850_10) and *KDR* rs1870377 (Assay ID: C__11895315_20) (all from Thermo Fisher Scientific, Waltham, MA, USA). Quality control included non-template controls in all runs and blind replicate assessment in 5% of the samples. Genotype was assigned for 96.3% of all samples tested.

### 2.4. Statistical Analysis

Descriptives were depicted as average ± standard deviation or median (interquartile range) according to departure from normality (Shapiro-Wilk test). Central tendency data was compared using the *t*-test, Mann-Whitney or Kruskal-Wallis tests according to normality and stratification of independent variables. Categorical variables were expressed as frequency (percentage) and proportions tested using the chi-square test. The difference in median months of PFS and OS from both cohorts was calculated through Kaplan-Meier curve with log rank test.

Four genetic models were considered in this study: each SNP was stratified according to the wild type allele into additive (“AA” genotype versus “Aa” versus “aa”), dominant (“AA” genotype versus “Aa” and “aa” genotypes), recessive (“Aa” and “AA” versus “aa” genotype) and heterosis (“Aa” versus “AA” and “aa” genotypes). The wild-type allele for each SNP was designated in agreement with Ensembl [37]. Empirical analyses were conducted on clinicopathological and genetic variables to test the association with time-to-outcome, using Cox regression analyses. After univariate analyses, *p* < 0.10 and an effect > 30% in hazard ratio (HR) were used as criteria for inclusion in a multivariated Cox with stepwise regression model. Genetic models with under-represented variant genotype (*n* < 4) were excluded from the multivariate analysis, and only the genetic model with the highest likelihood ratio for each SNP was included in the multivariate model to avoid collinearity. The resulting multivariated model was further tested with bootstrapping (MonteCarlo simulations, 1000 iterations), to elect the resulting relevant markers to be tested in the validation set. To investigate the possible predictive value of each SNP in treatment response for first line therapy, and time-to-progression or time-to-death, exploratory analyses were conducted examining the effect of genotype in a subgroup of patients treated with first line platinum doublet chemotherapy and in another subgroup of wild-type *EGFR* (epidermal growth factor receptor) patients, using the same survival analysis methods. Sample size, power, effect size and number of events for survival analyses (assuming alpha = 0.05 and power > 0.8) were estimated in both cohorts. Statistical analyses were performed on STATA software version 16.0 (StataCorp, College Station, TX, USA).

### 2.5. Ethics Approval

This project has been reviewed and approved by Coimbra University Hospital’s Ethical Committee (ref. no. 0111/CES) and by the Portuguese National Committee for data protection (ref. no. 2588/2017). The biobank protocol of Lungbiobank Heidelberg was approved by the ethics committee of the University of Heidelberg (S-270/2001).

## 3. Results

The clinicopathological characteristics of participating subjects from discovery and validation sets are described in Table 1. Regarding mutational status from discovery set (*n* = 167), we observed that 10.8% of patients (*n* = 18) had *EGFR* mutation (exon 19 deletions or exon 21 mutation), whereas 3.0% (*n* = 5) had rearrangements in the gene encoding anaplastic lymphocyte kinase (*ALK*). Mutations of *ROS1* were not detected. From the validation set (*n* = 190), 15.3% (*n* = 29) of NSCLC patients had *EGFR* mutation (exon 19 deletions or exon 21 mutation), 13.7% (*n* = 26) were *KRAS* mutated, 4.2% (*n* = 8) had *EML4-ALK* fusion, 2.6% (*n* = 5) had *BRAF* V600E mutation. No other targetable mutations were identified. Concerning respiratory co-morbidities from discovery set, twenty patients (12.0%) had moderate-severe COPD with lung emphysema and one patient (0.6%) had sarcoidosis, whereas in the validation set there were fifty patients (26.3%) with moderate-severe COPD and lung emphysema, and five patients (2.6%) had idiopathic pulmonary fibrosis.

The description of clinical-pathological variables is depicted in Table S2 with report of univariate analyses for PFS and OS. Genotypic distribution of functional SNPs in genes of the renin-angiotensin pathway, hypoxic and angiogenic factors using additive, dominant, recessive and heterosis models from discovery set are described in Table S3, together with hazard and survival univariate analyses. The univariate analysis showed that carriers of allele T (*n* = 112, 67.1%) for the SNP rs4316 in *ACE* gene had a significant protection to disease progression (Hazard ratio (HR) = 0.6, 95% confidence interval (CI) (0.4–0.9), *p* = 0.011). Homozygous GG/CC for *ACE2* rs908004 (*n* = 151, 90.4%) had increased risk for disease progression (HR = 2.1, 95% CI (1.1–3.9), *p* = 0.021). Another SNP from *MME* gene that encodes for neprilysin, showed a tendency between the dominant genetic model of *MME* rs701109 and PFS, having the heterozygous CT and homozygous CC protection to disease progression (HR = 0.6, 95% CI (0.3–1.0), *p* = 0.064). Univariate analysis also showed a significant association between the functional genetic variant of *VEGFA* rs25648 with time to tumour progression, being the homozygous C and T (*n* = 127, 78.9%) associated with worse prognosis (HR = 1.7, 95% CI (1.1–2.6), *p* = 0.030). The data showed a tendency between the heterosis genetic model of *KDR* rs1870377 and time to tumour progression, having the homozygous A and T increased risk to disease progression (HR = 1.4, 95% CI (1.0–2.0), *p* = 0.054) and to death (HR = 1.7, 95% CI (1.0–2.7), *p* = 0.034).

Multivariate analysis included the relevant variables (both clinical-pathological and genetic) from univariate empirical analysis (Table 2, Table 3). The C carriers (*n* = 149, 89.7%) for *MME* rs701109 had significantly decreased risk for disease progression than homozygotes T (*n* = 17, 10.3%), regardless of relevant clinical-pathological variables, namely tumour size, distant metastasis, type of systemic therapy, treatment modality or from other relevant SNPs (Table 2). Homozygous A and T for *KDR* rs1870377 (*n* = 99, 59.3%) had significantly lesser PFS and OS compared to heterozygous (*n* = 68, 40.7%), regardless of relevant clinical and pathological variables (Table 2 and Table 3). Notably, these results maintained its significance after boostrapping, as a statistical method for internal validation.

Exploratory subgroup analyses were conducted among NSCLC patients from discovery set that included only patients under first line platinum-based doublet chemotherapy without actionable driver mutations (*n* = 145) or *EGFR* wild-type (*n* = 149). Within the *MME* (rs701109) and *KDR* (rs1870377) polymorphisms, we found evidence for an independent effect at PFS and OS respectively, across all subgroups. Notably, CT and CC patients for *MME* (rs701109) under first line platinum-based doublet chemotherapy or *EGFR* wild-type had protection for disease progression (HR = 0.4, 95% CI (0.2–0.7), *p* = 0.005 and HR = 0.4, 95% CI (0.2–0.8), *p* = 0.006, respectively). Homozygous A and T for *KDR* rs1870377 had increased risk to death also in patients under first line platinum doublet chemotherapy or *EGFR* wild-type (HR = 1.9, 95% CI (1.1–3.2), *p* = 0.019 and HR = 2.0, 95% CI (1.2–3.4), *p* = 0.011, respectively). Moreover, from the multivariate analysis conducted in the subgroup of patients treated with first line platinum-based doublet chemotherapy without actionable driver mutations (*n* = 145), we also found that homozygous for *ACE2* rs908004 had increased risk for disease progression, regardless of relevant clinical-pathological covariates (HR = 2.9, 95% CI (1.3–6.3), *p* = 0.010).

The validation set included an independent matched cohort of NSCLC patients (Table 1) to validate the resulting genetic SNPs from the discovery set. Results from univariate analysis of clinicopathological variables in association with PFS and OS are described in Table S4. The genotype distribution and univariate analysis according to additive, dominant, recessive or heterosis models of *MME* rs701109, *KDR* rs1870377 and *ACE2* rs908004 for hazard and survival analysis are depicted in Table S5. The genetic distribution of genotypes in *MME* rs701109, *KDR* rs1870377 and *ACE2* rs908004 are similar among discovery and validation sets (*p* = 0.204, *p* = 0.846 and *p* = 0.251, respectively) (Pearson Chi-square test).

The multivariate model for PFS in the validation cohort is presented in Table 4. Homozygous (*n* = 102, 53.9%) for *MME* rs701109 had significantly increased risk to disease progression compared to CT genotype (*n* = 87, 46.0%), regardless of histology, type of systemic therapy or treatment modality (chemotherapy or chemo and radiotherapy). There was no significant effect on overall survival for *MME* rs701109, *KDR* rs1870377 and *ACE2* rs908004.

Subgroup analyses were also conducted in this set, for first line platinum-based doublet chemotherapy (*n* = 155) and *EGFR* wild-type patients (*n* = 161). The *MME* rs701109 polymorphism was associated with PFS only in the subgroup of patients with wild-type *EGFR*, being the homozygous at increased risk to disease progression (HR = 1.7, 95% CI (1.1–2.5), *p* = 0.009).

## 4. Discussion

The work developed here sought to test the hypothesis that variants in genes coding for the renin–angiotensin system and for interacting hypoxia/angiogenesis key molecules might have predictive and prognostic value, respectively for NSCLC progression and survival. SNPs are the most common type of polymorphisms, accounting for 90% of genetic variations [38]. Nucleotide substitution, according to its location in germline DNA, may elicit functional modifications, ultimately at protein level, contributing to inter-individual variability, and correlating with relevant phenotypic alterations. The association between genetic polymorphisms and cancer susceptibility, progression, aggressiveness and death has been thoroughly described in the literature [38].

RAS was first discovered and related with the physiological regulation of systemic arterial pressure and involved in hypertension pathogenesis [39]. As a gatekeeper system, RAS is responsible for more than one function, including cell cycle regulation, inflammation, angiogenesis and fibrosis [3]. The capillary blood vessels in the lung are one of the major sites of ACE expression and Ang II production in the human body [40]. In fact, the increased ACE expression observed in several interstitial lung diseases supports pulmonary RAS and establishes a putative role for Ang II in the response to lung injury and fibrosis [4]. A previous report showed that the G-6A polymorphism of the *AGT* gene is associated with increased angiotensin production and idiopathic pulmonary fibrosis progression [41], which is associated with increased risk of lung cancer [42]. Furthermore, the insertion/deletion (I/D) polymorphism of *ACE* gene has been linked to the pathogenesis and progression of human cancers [43,44] and the lowest serum ACE levels were correlated with poorer prognosis and higher relapse rates in lung cancer [15]. We found that the association between *ACE* rs4316 genetic variant and PFS, despite being significant in univariate, the finding was not confirmed on multivariate analysis. It is an I/D polymorphism and is localized at intron 16 of *ACE* gene. This SNP has been associated with plasma ACE levels, with DD genotype carriers independently associated to higher expression [45], albeit no effect was noted on AngII [45]. Thus, it seems unlikely this SNP might impact the ACE/AngII/ATR1 axis, which has been associated with tumour cell growth and angiogenesis, partially justifying the absence of clinical impact in NSCLC patients with advanced stage disease. There are other possible reasons for the failure to replicate the prior findings. Many of the initial reports were present only in retrospectively defined subsets of the subjects studied and the main hypotheses investigated were related to cancer risk. Another possibility is that varied ethnic background of study populations may result in a different genetic background which confers a different biologic significance to a given functional polymorphism.

ACE2 is responsible for converting AngII to Ang-(1-7), an agonist of Mas receptor (MasR). In a mouse lung xenograft model, it was previously shown that ACE2 overexpression inhibited both lung cancer cell proliferation In vitro and reduced tumour growth in vivo [46]. The *ACE2* rs908004 is an intronic variant at the X-linked *ACE2* gene [47]. Findings from the discovery cohort revealed that *ACE2* rs908004 heterozygous carriers presented shorter time-to-progression, only in the subgroup of patients treated with first line platinum-based chemotherapy without actionable driver mutations. We hypothesize that heterozygous produces reduced levels of ACE2, contributing to the up-regulation of ACE/Ang II/AT1 receptor axis, or to linkage with another genomic region that might be relevant for this clinical phenotype. These hypotheses need to be confirmed in further studies. Furthermore, as with all gene association studies, subgroup analyses can lead to spurious associations and might justify our result. Nevertheless, there is lack of studies on genotype–phenotype relationship with this SNP in lung cancer, opening new opportunities for further investigations.

Ang-(1-7) has opposite effects to Ang II upon binding to MasR and might be produced through hydroxylation of Ang I by neprilysin (NEP), an enzyme encoded by the gene *MME* [48]. NEP, also called CD10, has been described as a tumour-specific antigen for lymphocytic leukemia [49]. In the later years, it was demonstrated that NEP functions are implicated in oncogenesis and tumour microenvironment [49]. Moreover, alterations in NEP protein expression have also been reported in solid tumours, including lung cancer [50]. Both in discovery and validation cohorts, NSCLC subjects carrying the heterozygous TC genotype were at decreased risk for disease progression, albeit in the discovery set the protection was conferred to carriers of the TC/CC combined genotypes (dominant model), whereas in the validation cohort it was conferred only to TC (heterosis model). Heterosis for a single gene refers to a heterozygote that has a different phenotypic trait than for either allele homozygotes [51], and heterozygosity has been associated with human healthy aging [52]. Despite insufficient knowledge on the molecular basis of heterosis, an accepted mechanism is related to environmentally-driven epigenetic modifications, namely methylation and microRNAs (miRNAs) [53]. MicroRNAs regulate gene expression by binding to target sequences mostly at the 3’-UTR of mRNAs, and evidence exist for their functional effect in candidate SNPs of various types of cancer [54]. Noteworthy, recently reported data demonstrated such a mechanism with functional repercussion, the SNP rs6665, located downstream of rs701109 in the 3’-UTR region sequence of *MME* [55].

We conducted an *in-silico* proof-of-principle analysis using the MicroSNiPer database [56,57] to predict *MME* rs701109 SNP effects on putative microRNA targets, followed by study of the candidate miRNAs using the RNAhybrid program [58,59] to calculate the minimum free energy (MFE) of hybridization between miRNAs and target sequence (3′-UTR sequence of *MME* extracted from Ensembl [37]. The strongest binding affinity was found for hsa-miR-362-3p, which overlaps the locus of rs701109 at 3′-UTR region. A stronger predicted paring affinity between the hsa-miR-362-3p miRNA and the target mRNA of *MME* was observed for C-allele (−26.1 kcal/mol) in comparison to T-allele (−23.2 kcal/mol), stronger interaction with the miR-362-3p, resulting in lower expression of MME, and consecutively decreased risk for disease progression. Notably, previous data confirmed that hsa-miR-362-3p is expressed in lung tissue, aberrantly expressed in lung adenocarcinoma, and a promoter of metastasis [60] The differences among cohorts, particularly due to distinct therapeutics, genotype distribution and environmental stimuli, might account for different epigenetic regulation. Experimental studies in lung cancer tissues and In vitro with lung cancer organoids are warranted to further validate current findings.

The *MME* rs701109 variant consists in a nucleotide substitution of T-by-C, that has been shown to associate with lower NEP protein expression in the brain of patients with Alzheimer’s disease [61]. Notably, previous studies revealed that higher NEP expression associated with poorer prognosis in NSCLC and that tumour NEP was an independent predictor of recurrence in lung adenocarcinomas [50,62]. Taking into consideration that increased NEP expression in lung tumours associates with worst prognosis, and that TC and CC genotype carriers secrete less protein, our findings showing an independent protection for disease progression in TC carriers likely reflect the influence of this SNP in decreasing NEP availability. This result strengthens the involvement of NEP in NSCLC and supports the putative interest of including this genetic biomarker in a multilocus model for predicting relapse. Nevertheless, despite the known ability of NEP to degrade mitogenic peptides, the lack of Ang II degradation, as well as reduced production of Ang-(1-7) by NEP, might account for the growth of lung cancer cells [15]. Taken together, accumulated data and current findings support the need to foster research in neprilysin actions on cancer, particularly in lung cancer.

Hypoxia is a hallmark of solid tumours, and consequently related to enhanced aggressiveness, risk of disease progression and therapy resistance, effects known to be largely mediated by hypoxia-inducible factors [63]. The downstream upregulated vascular endothelial growth factor (VEGF) further contributes to cancer growth and metastasis, directly targeting tumour cells. Increased VEGF expression and/or circulating VEGF levels have been consistently reported in lung cancer [64]. Noteworthy, the activation of RAS in the tumour microenvironment usually involves Ang II, which has been described to induce hypoxia through reactive oxygen species production, and subsequent activation of pro-inflammatory and pro-angiogenic signals [65]. Albeit no clinical impact was demonstrated for polymorphisms in *HIFA*, *PGF* and *FLT1* genes, associations were observed in the discovery set for *VEGFA* rs25648 with PFS (univariate analysis) and *KDR* rs1870377 with PFS and OS (multivariate analysis). Koukourakis et al. [66], described that inherited *VEGF* sequence variations were strong determinants of the molecular VEGF and VEGF-downstream phenotype of NSCLC, being the substitution C > A at locus −2578 associated with higher levels of VEGF. Another study showed that *VEGFA* rs25648 (−73C > T) was a prognostic biomarker for PFS and OS in patients with hepatocellular carcinoma, with C-allele conferring improved prognosis, despite not confirmed in multivariate analysis [67]. In the discovery cohort, lung cancer patients had a different outcome since the homozygous C for *VEGF* rs25648 had lower time until disease progression in univariate analysis. The apparently conflicting results might be due to particularities related with different cancer models. Notably, studies investigating the potential association between *VEGF* polymorphisms and circulating VEGF levels also led to controversial results [68,69,70], opening new opportunities for further investigations to clarify the role of germline *VEGF* variations in NSCLC progression and its repercussion in VEGF levels.

The *KDR* rs1870377 was significantly associated with PFS and OS in the discovery set. Homozygous T and C had increased risk for tumour progression PFS and death, independent of clinicopathological variables and after bootstrap analysis. Findings were not replicated in the validation cohort. *KDR* encodes VEGFR-2, the key receptor mediating endothelial cell proliferation and sprouting, leading to angiogenesis and tumour promotion upon binding and signalling through the VEGFA pathway [71]. The *KDR* rs1870377 (c.1719A > T) variant induces a missense alteration in exon 11 that results in the substitution A > T and in the amino acid modification Gln472His. This substitution was previously described to alter the function of VEGF-KDR signalling pathway, enhancing ligand/receptor binding efficacy and upregulated signal transduction, ultimately leading to increased microvessel density [72]. This phenotypic correlation with the *KDR* rs1870377 might support T homozygous findings, hypothetically by promoting tumour vascular density for homozygous carriers. Previous findings reported that microvessel count, reflecting tumour neoangiogenesis, appeared to be a poor prognostic factor for survival in NSCLC patients [73], despite some conflicting results [74].

This study provides evidence that SNPs coding for molecules involved in the RAS (*ACE2* rs908004, *MME* rs701109, *KDR* rs1870377) might influence PFS and OS in advanced NSCLC patients, although only *MME* rs701109 was validated in an independent cohort. Candidate SNP studies searching associations with clinically relevant endpoints, have been reported to yield false-positive findings. Here, the multivariate model was first developed in a cohort of consecutively recruited advanced NSCLC patients. Furthermore, we included a robust data analyses setting that included: (i) sample size and number of events in both cohorts were within adequated estimations, (ii) initial empirical analyses to include only the most relevant variables in the multivariate models, (iii) bootstrapping using Montecarlo simulations with 1000 iterations to strengthen internal validation in each cohort, and (iv) validation of important SNPs from the discovery set in an independent cohort of lung cancer patients matched for demographic and pathological characteristics. Nevertheless, despite matching for clinicopathological characteristics, differences between discovery and validation cohorts existed, and this could partially explain the validation of a single polymorphism. Particularly, the validation set was selected from a larger lung cancer database and somatic mutational analyses were determined using distinct methods, revealing different mutational status for both cohorts, which likely influenced the course of disease and the subsequent therapeutic decisions. In addition, differences in respiratory co-morbidities, which may have conducted to mismatched treatment modalities (surgery with adjuvant chemotherapy versus chemo with radiotherapy for stages IIIA–IIIB), combined with the genetic background may also add to explain failure to validate the other genetic polymorphisms.

## 5. Conclusions

Germline genetic variants are common genomic events, putatively inducing phenotypic modifications affecting both normal physiology and cancer physiopathology. Data presented here supports the rationale for NEP and RAS mechanistic involvement in NSCLC, whereas the polymorphism *MME* rs701109 might prove to be a useful biomarker for NSCLC progression. These findings will foster further and larger studies to confirm the role of RAS, and particularly NEP in NSCLC, as well as to determine the usefulness of *MME* rs701109 as a molecular marker for disease progression. Taken together, these results can lead to a better understanding of NEP influence in the NSCLC biological mechanisms, contributing towards the definition of a genetic profile for this disease and eventually serve as guidance for therapeutic decisions.

## Figures and Tables

**Table 1:** Patient′s clinical characteristics.

Clinical Variables.	Discovery Set (*N* = 167)	Validation Set (*N* = 190)	*p*
Gender, *n*			
Males	123 (73.7%)	133 (70.0%)	
Females	44 (26.3%)	57 (30.0%)	0.445 *
Age (years)			
Median (IQR)	64.0 (58.0–72.0)	65.0 (58.0–73.0)	0.773 **
Smoking Status, *n* (%)			
Never smokers	31 (27.7%)	20 (10.5%)	
Previously smokers	68 (60.7%)	97 (51.1%)	
Smokers	13 (11.6%)	73 (38.4%)	<0.0001 *
Hypertension, *n* (%)			
Yes	67 (48.6%)	91 (47.9%)	
No	71 (51.4%)	99 (52.1%)	0.911 *
Anti-hypertension drugs, *n* (%)			
No	86 (65.2%)	126 (67.0%)	
iACE/ARB	46 (34.8%)	62 (33.0%)	0.728 *
ECOG PS, *n* (%)			
0–2	163 (97.6%)	188 (98.9%)	
>2	4 (2.4%)	2 (1.1%)	0.153 *
Histology, *n* (%)			
Adenocarcinoma	116 (69.5%)	126 (66.3%)	
Squamous cell carcinoma	42 (25.1%)	62 (32.6%)	
Adeno + Squamous cell	9 (5.4%)	2 (1.1%)	0.066 *
cTNM Stage, *n* (%)			
IIIA	20 (12.0%)	27 (14.2%)	
IIIB	32 (19.2%)	36 (18.9%)	
IIIC	21 (12.6%)	18 (9.5%)	
IVA	65 (38.9%)	66 (34.7%)	
IVB	29 (17.4%)	43 (22.6%)	0.597 *
Treatment modality, *n* (%)			
Surgery + ChT	12 (7.2%)	0 (0.0%)	
ChT + RT	31 (18.6%)	81 (42.6%)	
ChT	124 (74.3%)	109 (57.4%)	<0.0001 *
First-line treatment, *n* (%)			
Platinum based ChT	145 (86.8%)	155 (81.6%)	
TKI	22 (12.8%)	35 (18.4%)	0.194 *
PFS (months)			
Median, 95% CI	7.5 (6.1–9.0)	8.1 (6.8–9.4)	0.415 ***
OS (months)			
median, 95% CI	30.0 (16.9–43.2)	20.6 (16.3–24.9)	0.036 ***

IQR—interquartil range; iACE—inhibitors of angiotensin-converting enzyme, ARB—AngiotensinII receptor blocker, ECOG PS—Eastern cooperative Oncology Group Performance Status; ChT—chemotherapy; RT—radiotherapy; TKI—tyrosine kinase inhibitors; 95% CI—confidence interval. PFS—progression-free survival, OS—overall survival. * Pearson Chi Square test. ** Mann-Whitney U test. *** Kaplan Meier curve with log rank test.

**Table 2:** Multivariate analysis for progression-free survival (PFS) in the discovery cohort (*n* = 167).

Variables	PFS (Multivariate Stepwise Cox Proportional Hazards Model)		Bootstrap	
	HR (95% CI)	*p*	HR (95% CI)	*p*
T				
T1	Referent		Referent	
T2				
T3-4	1.7 (1.3–2.2)	<0.0001 *	1.7 (1.3–2.3)	<0.0001 *
Distant metastasis				
No	Referent		Referent	
Yes	1.7 (1.2–2.4)	0.005	1.7 (1.1–2.5)	0.009
Type of Therapy				
Surgery + ChT	Referent		Referent	
ChT				
ChT + RT	1.5 (1.1–2.1)	0.012 *	1.5 (1.0–2.3)	0.053 *
Type of Systemic Therapy, 1st line				
Platinum based ChT	Referent		Referent	
TKi	0.4 (0.2–0.8)	0.003	0.4 (0.2–0.7)	0.002
*KDR* rs1870377, Heterosis Model				
TA	Referent		Referent	
TT/AA	1.7 (1.2–2.5)	0.005	1.8 (1.2–7)	0.003
*MME* rs701109, Dominant Model				
TT	Referent		Referent	
TC/CC	0.5 (0.3–0.9)	0.018	0.5 (0.2–0.9)	0.030

Analyses were conducted using stepwise Cox regression, followed by Bootstrap analysis (Montecarlo simulations with 1000 iterations). HR, hazard ratio, 95% CI, confident interval, ChT, chemotherapy, RT, radiotherapy, TKi, tyrosine kinase inhibitors. * *p* trend value.

**Table 3:** Multivariate analysis of relevant variables for overall survival (OS) in the discovery set (*n* = 167) using stepwise Cox regression, followed by Bootstrap analysis (Montecarlo simulations with 1000 iterations).

Variables	OS (Multivariate Stepwise Cox Proportional Hazards Model)		Bootstrap	
	HR (95% CI)	*p*	HR (95% CI)	*p*
T				
T1	Referent		Referent	
T2				
T3-4	1.5 (1.1–2.2)	0.018 *	1.5 (1.0–2.2)	0.066 *
Distant metastasis				
No	Referent		Referent	
Yes	2.5 (1.5–4.3)	0.001	2.6 (1.5–4.5)	0.001
Age median				
≤64.0	Referent		Referent	
>64.0	1.7 (1.0–2.7)	0.031	1.6 (1.0–2.8)	0.069
*KDR* rs1870377, Heterosis Model				
TA	Referent		Referent	
TT/AA	2.1 (1.2–3.4)	0.006	2.0 (1.2–3.3)	0.012

HR, hazard ratio, 95% CI, confident interval. * *p* trend value.

**Table 4:** Multivariate analysis of relevant variables for disease progression using stepwise Cox regression, followed by Bootstrap analysis with 1000 iterations from validation set (*n* = 190).

Variables	PFS (Multivariate Stepwise Cox Proportional Hazards Model)		Bootstrap	
	HR (95%CI)	*p*	HR (95%CI)	*p*
Histology				
Adenocarcinoma	Referent		Referent	
Squamous cell	1.7 (1.2–2.4)	0.003 **	1.7 (1.2–2.5)	0.003 **
Other *	-		-	
Type of Therapy				
ChT	Referent		Referent	
ChT + RT	0.4 (0.3–0.6)	<0.0001	0.4 (0.3–0.6)	<0.0001
Type of Systemic Therapy, 1st line				
Platinum based ChT	Referent		Referent	
TKi	0.4 (0.3–0.7)	0.001	0.4 (0.3–0.7)	0.002
*MME* rs701109, Heterosis Model				
TC	Referent		Referent	
TT/CC	1.6 (1.2–2.3)	0.005	1.6 (1.1–2.4)	0.007

PFS—progression-free survival, HR—hazard ratio, 95% CI—confident interval, ChT—chemotherapy, RT—radiotherapy, TKi—inhibitors of tyrosine kinase. * Adenocarcinoma+squamous cell. ** *p* trend value.

## Supplementary Materials

The following are available online at https://www.mdpi.com/2072-6694/12/12/3834/s1, Table S1: Representative single nucleotide polymorphisms (SNPs) of genes involved in RAS (*ACE*, *ACE2*, *AGT*, *AGTR1*, *AGTR2*, *MME*, *CMA1*, *MAS1*) and SNPs of genes that express hypoxia-inducible factor 1-alpha, hypoxia-inducible factor 1-beta, hypoxia-inducible factor 2-alpha, hypoxia-inducible factor 2-beta, vascular endothelial growth factor and its receptor, placental growth factor and its receptor, Table S2. Clinical-pathological variables and progression-free survival (PFS) and overall survival (OS) in discovery set (*n* = 167)—Univariate analysis, Table S3: Discovery Set (*n* = 167): progression-free survival (PFS) and overall survival (OS)—Univariate analysis, Table S4. Clinical-pathological variables and progression-free survival (PFS) and overall survival (OS) in validation set (*n* = 190)—Univariate analysis, Table S5. Validation Set (*n* = 190)—Univariate analysis.
