# Supplementary material for: Germline Genetic Variants of the Renin-Angiotensin System, Hypoxia and Angiogenesis in Non-Small Cell Lung Cancer Progression: Discovery and Validation Studies
Source: Cancers (Basel). 2020 Dec 18;12(12):3834. doi: 10.3390/cancers12123834 (PMC7766842; doi:10.3390/cancers12123834)
Supplement: Supplementary file 1 [file cancers-12-03834-s001.pdf]

Table S1. Representative single nucleotide polymorphisms (SNPs) of genes involved in RAS (*ACE, ACE2, AGT, AGTR1, AGTR2, MME, CMA1, MAS1*) and SNPs of genes that express hypoxia-inducible factor 1-alpha, hypoxia-inducible factor 1-beta, hypoxia-inducible factor 2-alpha, hypoxia-inducible factor 2-beta, vascular endothelial growth factor and its receptor, placental growth factor and its receptor.

Genes	Single Nucleotide Polymorphisms
ACE	rs121912703, rs1799752, rs4340, rs4316, rs4317
ACE2	rs199951323, rs4646116, rs7890520, rs908004
AGT	rs699, rs34116584, rs121912923, rs4762
AGTR1	rs1800766, rs380400, rs5186, rs5185, rs5189, rs5187, rs12721226, rs12721225
AGTR2	rs11091046, rs5191, rs35474657, rs5194
MME	rs652438, rs701109, rs989692, rs777476150
CMA1	rs5250, rs7142020, rs140295781, rs201312283
MAS1	rs147267209, rs11968301, rs138263796, rs139579440
HIF1A	rs52801082, rs17834641, rs2057482, rs11549465, rs11549467
VEGFA	rs2010963, rs3025039, rs25648, rs10434, rs3025053, rs41282644
KDR	rs7667298, rs1870377, rs2305948
PGF	rs8185, rs12411, rs140032352, rs11542848
FLT1	rs2296283, rs7326277, rs9554314

Table S2. Clinical-pathological variables and progression-free survival (PFS) and overall survival (OS) in discovery set (n=167).

		PFS			OS		
	N	HR (95CI)	P *	Ptrend	HR (95CI)	P	Ptrend
Clinical variables							
**Age**							
**≤64.0**	85 (0.51)	Referent			Referent		
**>64.0**	82 (0.49)	0.9 (0.7-1.3)	0.678	-	1.6 (1.0-2.5)	0.047	-
**Gender**							
**Male**	123 (0.74)	Referent			Referent		
**Female**	44 (0.26)	0.9 (0.6-1.3)	0.539	-	0.6 (0.4-1.1)	0.105	-
**Histology**							
**Adenocarcinoma**	116 (0.70)	Referent			Referent		
**Squamous cell**	42 (0.25)	1.4 (0.9-2.1)	0.116		1.8 (1.1-3.0)	0.027	
**Others ***	9 (0.05)	0.8 (0.4-1.7)	0.571	0.680	0.9 (0.4-2.4)	0.902	0.291
**T**							
**1**	26 (0.16)	Referent			Referent		
**2**	46 (0.27)	1.3 (0.7-2.3)	0.339		0.5 (0.2-1.2)	0.138	
**3-4**	95 (0.57)	2.1 (1.3-3.6)	0.004	0.001	1.4 (0.8-2.7)	0.276	0.047
**N**							
**N0-N2**	63 (0.38)	Referent			Referent		
**N3**	103 (0.62)	1.4 (0.9-1.9)	0.099	-	1.1 (0.7-1.7)	0.815	-
**Distant Metastasis**							
**No**	73 (0.44)	Referent			Referent		
**Yes**	94 (0.56)	1.7 (1.2-2.4)	0.003	-	2.5 (1.5-4.2)	<0.001	
**Type_Therapy**							
**Surgery+ChT**	12 (0.07)	Referent			Referent		
**ChT**	124 (0.74)	2.9 (1.3-6.6)	0.012		2.7 (0.8-8.6)	0.099	
**ChT+RT**	31 (0.19)	3.1 (1.3-7.6)	0.011	0.026	3.0 (0.9-10.2)	0.080	0.127
**ECOG PS**							
**Good (0-2)**	163 (0.98)	Referent			Referent		
**Poor (3-4)**	4 (0.02)	1.4 (0.5-3.8)	0.501	-	2.1 (0.6-6.6)	0.223	-
**Anti-hypertensive drugs**							
**No**	86 (0.65)	Referent			Referent		
**iACE/ARB**	46 (0.35)	0.8 (0.6-1.3)	0.442	-	1.2 (0.7-2.1)	0.538	-

**Type_SysTher**							
**Platinum based**	145 (0.87)	Referent			Referent		
**TKI**	22 (0.13)	0.5 (0.3-0.9)	0.014	-	0.6 (0.3-1.4)	0.218	-

Univariate analysis. HR – Hazard Ratio. ChT – chemotherapy, RT – radiotherapy, TKI - tyrosine kinase inhibitor, iACE – inhibitors of angiotensin-converting enzyme, ARB – AngiotensinII receptor blocker.

Table S3. Discovery Set (n=167): progression-free survival (PFS) and overall survival (OS) – Univariate analysis.

			**Progression-free Survival**				**Overall Survival**		
**Genetic variants**	Genetic model	Genotype	N (freq.)	HR (95CI)	P *	Ptrend	HR (95CI)	P *	Ptrend
		CC	55 (0.33)	Referent			Referent		
***ACE* rs4316**	*Additive*	CT	82 (0.49)	0.6 (0.4-0.9)	0.013		0.6 (0.4-1.1)	0.089	
		TT	30 (0.18)	0.7 (0.4-1.1)	0.113	0.049	0.8 (0.4-1.2)	0.541	0.308
	Dominant	CC	55 (0.33)	Referent			Referent		
		CT/TT	112 (0.67)	0.6 (0.4-0.9)	**0.011**	-	0.7 (0.4-1.1)	0.114	
	Recessive	CC/CT	137 (0.82)	Referent			Referent		
		TT	30 (0.18)	0.9 (0.6-1.4)	0.676	-	1.0 (0.6-1.9)	0.896	
	Heterosis	CT	82 (0.49)	Referent			Referent		
		CC/TT	85 (0.51)	1.4 (1.0-2.0)	0.058	-	1.5 (0.9-2.3)	0.116	
***ACE* rs4317 ***		TT	167 (1.0)	-	-	-	**-**	**-**	
***ACE* rs121912703 ***		CC	167 (1.0)	-	-	-	**-**	**-**	
	Additive	GG	55 (0.33)	Referent			Referent		
***ACE2* rs908004**		GC	16 (0.09)	0.4 (0.2-0.9)	0.018		0.5 (0.2-1.1)	0.089	
		CC	96 (0.58)	0.9 (0.6-1.3)	0.566	0.794	0.9 (0.5-1.4)	0.587	0.681
	Dominant	GG	55 (0.33)	Referent			Referent		
		GC/CC	112 (0.67)	0.8 (0.6-1.2)	0.236	-	0.8 (0.5-1.3)	0.320	
	Recessive	GG/GC	71 (0.42)	Referent			Referent		
		CC	96 (0.58)	1.1 (0.8-1.6)	0.539	-	1.0 (0.7-1.7)	0.849	
	Heterosis	GC	16 (0.10)	Referent			Referent		
		GG/CC	151 (0.90)	2.1 (1.1-3.9)	**0.021**	-	2.0 (0.9-4.9)	0.109	
***ACE2* rs4646116 ***		TT	167 (1.0)	-	-	-	-	-	-
***ACE2* rs7890520**		GG	166 (0.99)	Referent					
		AG	1 (0.01)	-	-	-	-	-	-
ACE2* rs1999951323		A	167 (1.0)	-	-	-	-	-	-
***AGT* rs121912923 ***		GG	167 (1.0)	-	-	-	**-**	**-**	**-**
***AGT* rs4762**	*Additive*	GG	135 (0.81)	Referent			Referent		
		GA	31 (0.18)	1.0 (0.6-1.5)	0.964		0.8 (0.5-1.5)	0.495	0.495

		AA	1 (0.01)	-	-	-	-		
	Dominant	GG	135 (0.81)	Referent			Referent		
		GA/AA	32 (0.19)	1.0 (0.6-1.5)	0.917	-	0.8 (0.4-1.4)	0.389	
	Recessive	GG/GA	166 (0.99)	Referent			Referent		
		AA	1 (0.01)	-	-	-	2.0 (0.3-14.5)	0.492	
	Heterosis	GA	31 (0.19)	Referent			Referent		
		GG/GA	136 (0.81)	1.1 (0.7-1.7)	0.758	-	1.4 (0.7-2.7)	0.308	
***AGT* rs699**	*Additive*	GG	23 (0.14)	Referent			Referent		
		GA	87 (0.52)	0.8 (0.5-1.3)	0.370		1.6 (0.7-3.7)	0.303	
		AA	57 (0.34)	0.9 (0.5-1.6)	0.710	0.997	1.6 (0.7-4.0)	0.272	0.361
	Dominant	GG	23 (0.14)	Referent			Referent		
		GA/AA	144 (0.86)	0.8 (0.5-1.4)	0.464	-	1.6 (0.7-3.7)	0.271	
	Recessive	GG/GA	110 (0.66)	Referent			Referent		
		AA	57 (0.34)	1.1 (0.8-1.6)	0.622	-	1.1 (0.7-1.8)	0.634	
	Heterosis	AG	87 (0.52)	Referent			Referent		
		GG/AA	80 (0.48)	1.2 (0.8-1.7)	0.350	-	0.9 (0.6-1.5)	0.780	
***AGTR1* rs12721226 ***		GG	167 (1.0)	-	-	-	-	-	-
***AGTR1* rs1800766**	*Additive*	TT	115 (0.69)	Referent			Referent		
		TC	46 (0.27)	1.1 (0.7-1.6)	0.641		0.7 (0.4-1.3)	0.245	
		CC	6 (0.04)	1.0 (0.4-2.7)	0.999	0.726	0.4 (0.1-2.7)	0.372	0.135
	Dominant	TT	115 (0.69)	Referent			Referent		
		TC/CC	52 (0.31)	1.1 (0.7-1.6)	0.666	-	0.7 (0.4-1.2)	0.162	
	Recessive	TT/TC	161 (0.96)	Referent			Referent		
		CC	6 (0.04)	1.0 (0.4-2.6)	0.959	-	0.4 (0.1-2.9)	0.366	
	Heterosis	TC	46 (0.27)	Referent			Referent		
		TT/CC	121 (0.73)	0.9 (0.6-1.3)	0.639	-	1.4 (0.8-2.5)	0.284	
***AGTR1* rs5187 ***		AA	167 (1.0)	-	-	-	-	-	-
***AGTR1* rs380400**	*Additive*	GG	5 (0.03)	Referent			Referent		
		GA	31 (0.19)	0.4 (0.1-1.1)	0.067		0.3 (0.1-0.9)	0.037	
		AA	131 (0.78)	0.5 (0.2-1.3)	0.148	0.778	0.4 (0.1-1.2)	0.093	0.932
	Dominant	GG	5 (0.03)	Referent			Referent		
		GA/AA	162 (0.97)	0.5 (0.2-1.2)	0.124	-	0.3 (0.1-1.1)	0.073	
	Recessive	GG/GA	36 (0.22)	Referent			Referent		
		AA	131 (0.78)	1.2 (0.8-1.8)	0.455	-	1.2 (0.7-2.2)	0.524	
	Heterosis	AG	31 (0.19)	Referent			Referent		
		GG/AA	136 (0.81)	1.3 (0.8-2.1)	0.224	-	1.5 (0.8-2.8)	0.228	
***AGTR1* rs5186**	*Additive*	AA	87 (0.52)	Referent			Referent		
		AC	66 (0.40)	1.0 (0.7-1.5)	0.901		0.9 (0.6-1.5)	0.755	

		CC	14 (0.08)	1.3 (0.7-2.6)	0.370	0.513	1.9 (0.8-4.2)	0.131	0.461
	Dominant	AA	87 (0.52)	Referent			Referent		
		AC/CC	80 (0.48)	1.1 (0.8-1.5)	0.715	-	1.0 (0.6-1.6)	0.892	
	Recessive	AA/AC	153 (0.92)	Referent			Referent		
		CC	14 (0.08)	1.3 (0.7-2.5)	0.371	-	1.9 (0.9-4.3)	0.102	
	Heterosis	AC	66 (0.39)	Referent			Referent		
		AA/CC	101 (0.61)	1.0 (0.7-1.4)	0.939		1.2 (0.7-1.9)	0.535	
***AGTR1* rs5185**		TT	164 (0.98)	Referent					
		TG	3 (0.02)	0.2 (0.0-1.4)	0.102	-	-	-	-
***AGTR1* rs5189**	*Additive*	TT	1 (0.01)	Referent			Referent		
		TG	12 (0.07)	-	-		-		
		GG	154 (0.92)	-	-	-	1.2 (0.4-3.4)	0.665	0.665
	Dominant	TT	1 (0.01)	Referent			Referent		
		TG/GG	166 (0.99)	-	-	-	.	-	
	Recessive	TT/TG	13 (0.08)	Referent			Referent		
		GG	154 (0.92)	1.0 (0.5-1.8)	0.928	-	1.7 (0.5-5.4)	0.375	
	Heterosis	GT	12 (0.07)	Referent			Referent		
		TT/GG	155 (0.93)	1.1 (0.5-2.1)	0.817	-	2.6 (0.6-10.5)	0.190	
***AGTR2* rs5191 ***		GG	167 (1.0)	-	-	-	-	-	-
***AGTR2* rs5194**	*Additive*	GG	85 (0.51)	Referent			Referent		
		GA	20 (0.12)	1.1 (0.6-1.9)	0.795		1.1 (0.5-2.3)	0.801	
		AA	62 (0.37)	1.0 (0.7-1.5)	0.856	0.848	0.9 (0.5-1.5)	0.692	0.706
	Dominant	GG	85 (0.51)	Referent			Referent		
		AG/AA	82 (0.49)	1.0 (0.7-1.5)	0.804	-	0.9 (0.6-1.5)	0.815	
	Recessive	GG/AG	105 (0.63)	Referent			Referent		
		AA	62 (0.37)	1.0 (0.7-1.5)	0.910	-	0.9 (0.5-1.4)	0.628	
	Heterosis	AG	20 (0.12)	Referent			Referent		
		GG/AA	147 (0.88)	0.9 (0.6-1.6)	0.828	-	0.9 (0.4-1.7)	0.702	
***AGTR2* rs11091046**	*Additive*	AA	62 (0.37)	Referent			Referent		
		AC	20 (0.12)	1.0 (0.6-1.8)	0.893		1.2 (0.6-2.6)	0.615	
		CC	85 (0.51)	1.0 (0.7-1.4)	0.856	0.848	1.1 (0.7-1.8)	0.692	0.706
	Dominant	AA	62 (0.37)	Referent			Referent		
		AC/CC	105 (0.63)	1.0 (0.7-1.4)	0.910	-	1.1 (0.7-1.8)	0.628	
	Recessive	AA/AC	82 (0.49)	Referent			Referent		
		CC	85 (0.51)	1.0 (0.7-1.3)	0.804	-	1.1 (0.7-1.7)	0.815	
	Heterosis	AC	20 (0.12)	Referent			Referent		
		AA/CC	147 (0.88)	0.9 (0.6-1.6)	0.828	-	0.9 (0.4-1.8)	0.702	
***AGTR2* rs35474657 ***		AA	167 (1.0)	-	-	-	-	-	-

***MAS1* rs11968301 ***		AA	167 (1.0)	-	-	-	-	-	-
***MAS1* rs138263796 ***		GG	167 (1.0)	-	-	-	-	-	-
***MAS1* rs139579440 ***		CC	167 (1.0)	-	-	-	-	-	-
***MAS1* rs147267209 ***		AA	167 (1.0)	-	-	-	-	-	-
	Additive	GG	55 (0.33)	Referent			Referent		
***VEGFA* rs10434**		GA	81 (0.48)	1.1 (0.8-1.7)	0.486		1.0 (0.6-1.6)	0.851	
		AA	31 (0.19)	1.3 (0.8-2.2)	0.238	0.236	1.1 (0.6-2.1)	0.718	0.785
	Dominant	GG	55 (0.33)	Referent			Referent		
		GA/AA	112 (0.67)	1.2 (0.8-1.7)	0.332	-	1.0 (0.6-1.6)	0.985	
	Recessive	GG/GA	136 (0.81)	Referent			Referent		
		AA	31 (0.19)	1.2 (0.8-1.9)	0.330	-	1.2 (0.7-2.0)	0.626	
	Heterosis	GA	81 (0.48)	Referent			Referent		
		GG/AA	86 (0.52)	1.0 (0.7-1.4)	0.830	-	1.1 (0.7-1.8)	0.712	
	Additive	GG	67 (0.40)	Referent			Referent		
***VEGFA* rs2010963**		GC	73 (0.44)	1.2 (0.8-1.7)	0.436		1.2 (0.7-2.0)	0.459	
		CC	27 (0.16)	1.5 (1.0-2.5)	0.088	0.099	1.0 (0.5-1.9)	0.886	0.895
	Dominant	GG	67 (0.40)	Referent			Referent		
		GC/CC	100 (0.60)	1.2 (0.9-1.8)	0.213	-	1.1 (0.7-1.8)	0.608	
	Recessive	GG/GC	140 (0.84)	Referent			Referent		
		CC	27 (0.16)	1.4 (0.9-2.2)	0.125	-	0.9 (0.5-1.7)	0.667	
	Heterosis	GC	73 (0.44)	Referent			Referent		
		GG/CC	94 (0.56)	1.0 (0.7-1.4)	0.868	-	0.8 (0.5-1.3)	0.394	
	Additive	CC	122 (0.76)	Referent			Referent		
***VEGFA* rs25648**		CT	34 (0.21)	0.6 (0.4-0.9)	0.029		1.0 (0.5-1.7)	0.913	
		TT	5 (0.03)	0.9 (0.3-2.5)	0.837	0.072	1.9 (0.6-6.2)	0.277	0.603
	Dominant	CC	122 (0.76)	Referent			Referent		
		CT/TT	39 (0.24)	0.6 (0.4-1.0)	**0.036**	-	1.1 (0.6-1.8)	0.838	
	Recessive	CC/CT	156 (0.97)	Referent			Referent		
		TT	5 (0.03)	1.0 (0.4-2.7)	0.997	-	1.9 (0.6-6.2)	0.269	
	Heterosis	CT	34 (0.21)	Referent			Referent		
		CC/TT	127 (0.79)	1.7 (1.1-2.6)	**0.030**	-	1.1 (0.6-1.9)	0.847	
	Additive	CC	123 (0.74)	Referent			Referent		
***VEGFA* rs3025039**		CT	42 (0.25)	1.0 (0.7-1.5)	0.768		0.8 (0.4-1.4)	0.545	-
		TT	2 (0.01)	-	-	-	-	-	-
	Dominant	CC	123 (0.74)	Referent			Referent		
		CT/TT	44 (0.26)	1.0 (0.7-1.5)	0.940	-	0.8 (0.5-1.4)	0.433	
	Recessive	CC/CT	165 (0.99)	Referent			Referent		
		TT	2 (0.01)	2.4 (0.6-9.8)	0.225	-	2.5 (0.3-17.9)	0.373	

	Heterosis	CT	42 (0.25)	Referent			Referent		
		CC/TT	125 (0.75)	1.0 (0.7-1.5)	0.875	-	1.3 (0.7-2.3)	0.343	
***VEGFA* rs41282644**		GG	153 (0.92)	Referent			Referent		
		GA	14 (0.08)	1.3 (0.7-2.4)	0.454	-	1.0 (0.5-2.4)	0.910	-
***KDR* rs2305948**	*Additive*	CC	123 (0.74)	Referent			Referent		
		CT	40 (0.24)	1.0 (0.7-1.5)	0.903		1.4 (0.8-2.4)	0.186	
		TT	4 (0.02)	0.7 (0.3-2.0)	0.508	0.735	0.3 (0.4-2.2)	0.239	0.974
	Dominant	CC	123 (0.74)	Referent			Referent		
		CT/TT	44 (0.26)	1.0 (0.7-1.4)	0.913	-	1.2 (0.7-2.0)	0.488	
	Recessive	CC/CT	163 (0.98)	Referent			Referent		
		TT	4 (0.02)	0.7 (0.3-1.9)	0.498	-	0.3 (0.0-2.0)	0.211	
	Heterosis	CT	40 (0.24)	Referent			Referent		
		CC/TT	127 (0.76)	1.0 (0.6-1.4)	0.841	-	0.7 (0.4-1.1)	0.145	
***KDR* rs1870377**	*Additive*	TT	93 (0.56)	Referent			Referent		
		TA	68 (0.40)	0.7 (0.5-1.0)	0.082		0.6 (0.4-1.0)	0.064	
		AA	6 (0.04)	1.9 (0.8-4.8)	0.163	0.347	4.1 (1.4-11.8)	0.010	0.517
	Dominant	TT	93 (0.56)	Referent			Referent		
		TA/AA	74 (0.44)	0.8 (0.5-1.1)	0.143	-	0.7 (0.4-1.1)	0.160	
	Recessive	TT/TA	161 (0.96)	Referent			Referent		
		AA	6 (0.04)	2.2 (0.9-5.5)	0.088	-	4.9 (1.7-14.0)	0.003	
	Heterosis	TA	68 (0.41)	Referent			Referent		
		TT/AA	99 (0.59)	1.4 (1.0-2.0)	**0.054**	-	1.7 (1.0-2.7)	**0.034**	
***KDR* rs7667298**	*Additive*	TT	24 (0.14)	Referent			Referent		
		TC	84 (0.51)	1.1 (0.6-1.8)	0.806		1.5 (0.7-3.0)	0.274	
		CC	57 (0.35)	1.0 (0.6-1.7)	0.927	0.990	0.9 (0.4-2.0)	0.859	0.482
	Dominant	TT	24 (0.14)	Referent			Referent		
		TC/CC	141 (0.86)	1.0 (0.6-1.7)	0.849	-	1.2 (0.6-2.3)	0.600	
	Recessive	TT/TC	108 (0.65)	Referent			Referent		
		CC	57 (0.35)	1.0 (0.7-1.4)	0.899	-	0.7 (0.4-1.1)	0.155	
	Heterosis	TC	84 (0.51)	Referent			Referent		
		TT/CC	81 (0.49)	1.0 (0.7-1.3)	0.793	-	0.6 (0.4-1.0)	0.067	
***FLT1* rs2296283**	*Additive*	GG	32 (0.19)	Referent			Referent		
		GA	80 (0.48)	1.4 (0.9-2.3)	0.149		1.4 (0.7-2.7)	0.378	
		AA	55 (0.33)	1.2 (0.7-1.9)	0.583	0.785	1.2 (0.6-2.5)	0.638	0.810
	Dominant	GG	32 (0.19)	Referent			Referent		
		GA/AA	135 (0.81)	1.3 (0.8-2.1)	0.245	-	1.3 (0.7-2.5)	0.445	-
	Recessive	GG/GA	112 (0.67)	Referent			Referent		
		AA	55 (0.33)	0.9 (0.6-1.3)	0.564	-	0.9 (0.6-1.5)	0.798	-

	Heterosis	GA	80 (0.48)	Referent			Referent		
		GG/AA	87 (0.52)	0.8 (0.5-1.1)	0.136	-	0.8 (0.5-1.3)	0.415	-
***FLT1* rs9554314**		AC	34 (0.20)	Referent			Referent		
		AA	133 (0.80)	0.7 (0.5-1.1)	0.119	-	0.7 (0.4-1.2)	0.167	-
***FLT1* rs7326277**		TT	133 (0.80)	Referent			Referent		
		TC	34 (0.20)	1.4 (0.9-2.1)	0.119	-	1.4 (0.9-2.4)	0.167	-
***HIF1A* rs11549465**	*Additive*	CC	124 (0.74)	Referent			Referent		
		CT	42 (0.25)	1.0 (0.7-1.5)	0.875		1.4 (0.9-2.4)	0.164	
		TT	1 (0.01)	-	-	-	-	-	-
	Dominant	CC	124 (0.74)	Referent			Referent		
		CT/TT	43 (0.26)	0.9 (0.6-1.4)	0.699	-	1.3 (0.8-2.2)	0.251	
	Recessive	CC/CT	166 (0.99)	Referent			Referent		
		TT	1 (0.01)	-	-	-	-	-	
	Heterosis	CT	41 (0.25)	Referent			Referent		
		CC/TT	126 (0.75)	1.0 (0.7-1.5)	0.966	-	0.7 (0.4-1.1)	0.144	
***HIF1A* rs11549467 ***		GG	167 (1.0)	-	-	-	-	-	-
***HIF1A* rs2057482**	*Additive*	TT	6 (0.04)	Referent			Referent		
		TC	53 (0.31)	1.6 (0.6-4.4)	0.384		1.3 (0.3-5.5)	0.729	
		CC	108 (0.65)	1.6 (0.6-4.4)	0.355	0.524	1.0 (0.2-4.0)	0.966	0.346
	Dominant	TT	6 (0.04)	Referent			Referent		
		TC/CC	161 (0.96)	1.6 (0.6-4.3)	0.357	-	1.1 (0.3-4.4)	0.925	
	Recessive	TT/TC	59 (0.35)	Referent			Referent		
		CC	108 (0.65)	1.1 (0.7-1.5)	0.718	-	0.8 (0.5-1.2)	0.268	
	Heterosis	TC	53 (0.31)	Referent			Referent		
		TT/CC	114 (0.69)	1.0 (0.7-1.4)	0.964	-	0.8 (0.5-1.2)	0.243	
***HIF1A* 52801082**	*Additive*	CC	124 (0.74)	Referent			Referent		
		CT	41 (0.25)	1.0 (0.7-1.5)	0.875		1.4 (0.9-2.4)	0.164	
		TT	2 (0.01)	-	-	-	-	-	-
	Dominant	CC	124 (0.74)	Referent			Referent		
		CT/TT	43 (0.26)	0.9 (0.6-1.4)	0.699	-	1.3 (0.8-2.2)	0.251	
	Recessive	CC/CT	165 (0.99)	Referent			Referent		
		TT	2 (0.01)	0.3 (0.0-2.2)	0.241	-	-	-	
	Heterosis	CT	41 (0.25)	Referent			Referent		
		CC/TT	126 (0.75)	1.0 (0.7-1.5)	0.966	-	0.7 (0.4-1.1)	0.144	
***PGF* rs12411**	*Additive*	TT	6 (0.04)	Referent			Referent		
		TA	44 (0.26)	1.8 (0.7-4.7)	0.206		1.4 (0.5-4.0)	0.587	
		AA	117 (0.70)	1.5 (0.6-3.8)	0.347	0.913	1.2 (0.4-3.5)	0.691	0.937

	Dominant	TT	6 (0.04)	Referent			Referent		
		TA/AA	161 (0.96)	1.6 (0.7-4.0)	0.296	-	1.3 (0.5-3.5)	0.654	
	Recessive	TT/TA	50 (0.30)	Referent			Referent		
		AA	117 (0.70)	0.9 (0.6-1.3)	0.687	-	1.0 (0.6-1.6)	0.891	
	Heterosis	TA	44 (0.26)	Referent			Referent		
		TT/AA	123 (0.74)	0.8 (0.6-1.2)	0.309	-	0.9 (0.5-1.5)	0.683	
***PGF* rs140032352 ***		GG	167 (1.00)	-	-	-	-	-	-
	Additive	TT	147 (0.88)	Referent			Referent		
***PGF* rs8185**		TC	16 (0.10)	1.1 (0.6-1.9)	0.845		1.3 (0.6-2.7)	0.472	
		CC	4 (0.02)	0.6 (0.2-1.9)	0.363	0.544	0.6 (0.2-2.6)	0.517	0.889
	Dominant	TT	147 (0.88)	Referent			Referent		
		TC/CC	20 (0.12)	0.9 (0.5-1.6)	0.756	-	1.1 (0.6-2.1)	0.828	
	Recessive	TT/TC	163 (0.98)	Referent			Referent		
		CC	4 (0.02)	0.6 (0.2-1.8)	0.358	-	0.6 (0.1-2.5)	0.495	
	Heterosis	TC	16 (0.10)	Referent			Referent		
		TT/CC	151 (0.90)	0.9 (0.5-1.6)	0.800	-	0.8 (0.4-1.6)	0.446	
***CMA1* rs140295781 ***		CC	167	-	-	-	-	-	-
***CMA1* rs201312283 ***		GG	167	-	-	-	-	-	-
	Additive	GG	129 (0.77)	Referent			Referent		
***CMA1* rs5250**		GA	36 (0.22)	0.9 (0.6-1.3)	0.565		1.1 (0.6-1.8)	0.792	
		AA	2 (0.01)	-	-	-	-	-	-
	Dominant	GG	129 (0.77)	Referent			Referent		
		GA/AA	38 (0.23)	0.8 (0.6-1.3)	0.412	-	1.0 (0.6-1.7)	0.921	
	Recessive	GG/GA	165 (0.99)	Referent			Referent		
		AA	2 (0.01)	0.8 (0.2-3.4)	0.805	-	0.6 (0.1-4.0)	0.563	
	Heterosis	GA	36 (0.22)	Referent			Referent		
		GG/AA	131 (0.78)	1.2 (0.8-1.8)	0.447	-	0.9 (0.5-1.6)	0.752	
***CMA1* rs7142020 ***		AA	167 (1.0)	-	-	-	-	-	-
***MME* rs777476150 ***		CC	167 (1.0)	-	-	-	-	-	-
	Additive	TT	141 (0.85)	Referent			Referent		
***MME* rs652438**		TC	24 (0.14)	1.4 (0.9-2.2)	0.178		1.4 (0.8-2.7)	0.266	
		CC	2 (0.01)	-	-	-	-	-	-
	Dominant	TT	141 (0.85)	Referent			Referent		
		CT/CC	26 (0.15)	1.2 (0.8-1.9)	0.404	-	1.4 (0.7-2.5)	0.304	
	Recessive	TT/TC	165 (0.99)	Referent			Referent		
		CC	2 (0.01)	0.3 (0.0-2.3)	0.259	-	0.9 (0.1-6.5)	0.917	
	Heterosis	TC	24 (0.14)	Referent			Referent		
		TT/CC	143 (0.86)	0.7 (0.4-1.1)	0.142	-	0.7 (0.4-1.3)	0.264	

MME **rs701109**	*Additive*	TT	17 (0.10)	Referent			Referent		
		CT	72 (0.44)	0.6 (0.3-1.2)	0.134		0.7 (0.3-1.4)	0.277	
		CC	77 (0.46)	0.5 (0.3-0.9)	0.045	0.074	0.6 (0.3-1.3)	0.189	0.271
	Dominant	TT	17 (0.10)	Referent			Referent		
		TC/CC	149 (0.90)	0.6 (0.3-1.0)	**0.064**	-	0.6 (0.3-1.3)	0.203	
	Recessive	TT/TC	89 (0.54)	Referent			Referent		
		CC	77 (0.46)	0.8 (0.6-1.1)	0.200	-	0.8 (0.5-1.4)	0.489	
	Heterosis	TC	72 (0.43)	Referent			Referent		
		CC/TT	94 (0.57)	0.9 (0.7-1.3)	0.688	-	1.0 (0.6-1.6)	0.987	
MME **rs989692**	*Additive*	TT	39 (0.23)	Referent			Referent		
		TC	69 (0.42)	0.9 (0.6-1.4)	0.669		1.4 (0.7-2.5)	0.327	
		CC	58 (0.35)	0.8 (0.5-1.3)	0.364	0.358	1.3 (0.7-2.5)	0.385	0.421
	Dominant	TT	39 (0.23)	Referent			Referent		
		TC/CC	127 (0.77)	0.9 (0.6-1.3)	0.473	-	1.3 (0.8-2.4)	0.306	
	Recessive	TT/TC	108 (0.65)	Referent			Referent		
		CC	58 (0.35)	0.9 (0.6-1.2)	0.419	-	1.1 (0.7-1.8)	0.729	
	Heterosis	TC	69 (0.42)	Referent			Referent		
		TT/CC	97 (0.58)	1.0 (0.7-1.4)	0.863	-	0.9 (0.5-1.4)	0.557	

* SNPs without variation in this population. HR, hazard ratio; 95%CI, 95% confidence interval. After univariate Cox regression analyses, the values for retention were $P<0.10$ and more or less than 30% effect in HR, in order to be included into the multivariate Cox proportional hazards model. Only one genetic model was included in the model to avoid collinearity. Tag SNPs: *AGTR2* (rs5194, rs11091046), *FLT1* (rs9554314, rs11549465), *HIF1A* rs52801082, *KDR* (rs1531289, rs2239702, rs7667298, rs2305948, rs1870377).

Table S4. Clinical-pathological variables and progression-free survival (PFS) and overall survival (OS) in validation set (n=190).

		PFS			OS		
		Univariate (Cox)			Univariate (Kaplan-Meier)		
	N	HR (95CI)	P *	Ptrend	HR (95CI)	P *	Ptrend
**Clinical variables**							
**Age**							
**<65.0**	89 (0.47)	Referent		-	Referent	**0.027**	-
**>65.0**	101 (0.53)	1.3 (0.9-1.8)	0.133		1.6 (1.0-2.5)		
**Gender**							
**Male**	133 (0.70)	Referent	**0.015**	-	Referent	0.586	-
**Female**	57 (0.30)	0.6 (0.4-0.9)			0.9 (0.6-1.3)		
**Histology**							
**Adenocarcinoma**	126 (0.66)	Referent		**0.006**	Referent		**0.066**
**Squamous cell**	62 (0.33)	1.5 (1.1-2.1)	0.14		1.7 (1.1-2.2)	**0.021**	
**Others ***	2 (0.01)	3.2 (0.8-13.3)	0.103		-	**-**	
**T**							
**T1**	16 (0.08)	Referent		0.595	Referent		0.308
**T2**	33 (0.18)	1.3 (0.6-2.7)	0.485		1.6 (0.5-4.9)	0.424	
**T3/T4**	141 (0.74)	1.3 (0.7-2.4)	0.473		1.7 (0.6-4.7)	0.288	
**N**							
**N0-N2**	114 (0.60)	Referent	0.268	-	Referent	0.376	-
**N3**		1.2 (0.9-1.7)			1.2 (0.8-1.8)		

	76 (0.40)						
**Distant Metastasis**							
**No**	81 (0.43)	Referent	0.085	-	Referent	0.669	-
**Yes**	109 (0.57)	1.3 (1.0-1.9)			1.1 (0.7-1.7)		
**ECOG PS**							
**Good (0-2)**	188 (0.99)	Referent	**<0.0001**	-	Referent	**<0.0001**	-
**Poor (3-4)**	2 (0.01)	18.9 (4.1-86.3)			22.3 (5.0-100.9)		
**Type_Therapy**							
**ChT**	109 (0.57)	Referent	0.052	-	Referent	0.163	-
**ChT+RT**	81 (0.43)	0.7 (0.5-1.0)			0.7 (0.5-1.1)		
**Type_SysTher**							
**Platinum based ChT**	155 (0.82)	Referent	**0.003**	-	Referent	**0.020**	-
**TKI**	35 (0.18)	0.5 (0.3-0.8)			0.5 (0.3-0.9)		
**Anti-hypertensive drugs**							
**No**	126 (0.67)	Referent	0.441	-	Referent	0.892	-
**iACE/ARB**	62 (0.33)	1.1 (0.8-1.6)			1.0 (0.6-1.5)		

Univariate analysis. HR – Hazard Ratio. ChT – chemotherapy, RT – radiotherapy, TKI - tyrosine kinase inhibitor, iACE – inhibitors of angiotensin-converting enzyme, ARB – AngiotensinII receptor blocker

Table S5. Validation Set (n=190).

				Progression-free survival Univariate (Cox)			Overall survival Univariate (Cox)		
**Genetic variants**	Genetic model		N	HR (95CI)	P *	Ptrend	HR (95CI)	P	Ptrend
ACE2	*Additive*	G	61 (0.32)	Referent			Referent		
rs908004		GC	29 (0.15)	1.1 (0.6-1.7)	0.819	0.425	0.8 (0.4-1.5)	0.443	
		C	99 (0.53)	0.9 (0.6-1.2)	0.459		0.8 (0.5-1.2)	0.281	0.299
	Dominant	G	61 (0.32)	Referent	0.587	-	Referent	0.256	
		GC/C	128 (0.68)	0.9 (0.6-1.3)			0.8 (0.5-1.2)		-
	Recessive	G/GC	90 (0.48)	Referent	0.350	-	Referent	0.421	
		C	99 (0.52)	0.8 (0.6-1.2)			0.8 (0.6-1.3)		-
	Heterosis	GC	29 (0.15)	Referent	0.531	-	Referent	0.735	
		G/C	160 (0.85)	0.9 (0.6-1.4)			1.1 (0.6-2.0)		-
KDR	*Additive*	TT	108 (0.58)	Referent			Referent		
rs1870377		TA	74 (0.39)	0.9 (0.7-1.4)	0.932	0.958	1.1 (0.7-1.7)	0.674	
		AA	5 (0.03)	1.0 (0.3-3.3)	0.959		1.2 (0.3-5.1)	0.632	0.632
	Dominant	TT	108 (0.58)	Referent	0.941	-	Referent	0.652	
		TA/AA	79 (0.42)	1.0 (0.7-1.4)			1.1 (0.7-1.7)		-
	Recessive	TT/TA	182 (0.97)	Referent	0.951	-	Referent	0.815	
		AA	5 (0.03)	1.0 (0.3-3.3)			1.2 (0.3-4.8)		-
	Heterosis	TA	74 (0.40)	Referent	0.927	-	Referent	0.699	
		TT/AA	113 (0.60)	1.0 (0.7-1.4)			0.9 (0.6-1.4)		-
MME	*Additive*	TT	29 (0.15)	Referent			Referent		
rs701109		TC	87 (0.46)	0.7 (0.4-1.1)	0.115	0.665	0.8 (0.4-1,4)	0.381	
		CC	73 (0.39)	1.0 (0.6-1.5)	0.882		1.0 (0.6-1.8)	0.954	0.698

	Dominant	TT	29 (0.15)	Referent	0.308	-	Referent	0.628	
		TC/CC	160 (0.85)	0.8 (0.5-1.2)			0.9 (0.5-1.5)		-
	Recessive	TT/TC	116 (0.61)	Referent			Referent		
		CC	73 (0.39)	1.3 (0.9-1.8)	0.165		1.2 (0.8-1.9)	0.347	-
	Heterosis	TC	87 (0.46)	Referent			Referent		
		TT/CC	102 (0.54)	1.4 (1.0-1.9)	0.041	-	1.3 (0.8-2.0)	0.207	-

Univariate analysis. HR, hazard ratio; 95%CI, 95% confidence interval. After univariate Cox regression analyses, the values for retention were P<0.10 and more or less than 30% effect in HR, in order to be included into the multivariate Cox proportional hazards model. Only one genetic model was included in the model to avoid collinearit
